# Ontogeny of Hypothalamus-Pituitary Gonadal Axis and Minipuberty: An Ongoing Debate?

**DOI:** 10.3389/fendo.2020.00187

**Published:** 2020-04-07

**Authors:** Carla Bizzarri, Marco Cappa

**Affiliations:** Unit of Endocrinology, Bambino Gesù Children's Hospital (IRCCS), Rome, Italy

**Keywords:** minipuberty, hypothalamus-pituitary-gonadal axis, infant, testosterone, estrogen

## Abstract

The fetal hypothalamus-pituitary gonadal (HPG) axis begins to function during mid-gestation but its activity decreases during late pregnancy due to the suppressive effects of placental estrogens. Placental hormones drop immediately after birth, FSH and LH surge at around 1 week and peak between 1 and 3 months of life. The HPG axis is activated in both sexes, but a sexual dimorphism is evident with higher LH values in boys, while FSH prevails in girls. Both gonadotrophins decline in boys by around 6 months of age. In girls, LH declines at the same time as in boys, while FSH persists elevated up to 3 or 4 years of age. As a result of gonadotropin activation, testicular testosterone increases in males and ovarian estradiol rises in females. These events clinically translate into testicular and penile growth in boys, enlargement of uterus and breasts in girls. The functional impact of HPG axis activity in infancy on later reproductive function is uncertain. According to the perinatal programming theory, this period may represent an essential programming process. In boys, long-term testicular hormonal function and spermatogenesis seem to be, at least in part, regulated by minipuberty. On the contrary, the role of minipuberty in girls is still uncertain. Recently, androgen exposure during minipuberty has been correlated with later sex-typed behavior. Premature and/or SGA infants show significant differences in postnatal HPG axis activity in comparison to full-term infants and the consequences of these differences on later health and disease require further research. The sex-dimorphic HPG activation during mid-gestation is probably responsible for the body composition differences observed ad birth between boys and girls, with boys showing greater total body mass and lean mass, and a lower proportion of fat mass. Testosterone exposure during minipuberty further contributes to these differences and seems to be responsible for the significantly higher growth velocity observed in male infants. Lastly, minipuberty is a valuable “window of opportunity” for differential diagnosis of disorders of sex development and it represents the only time window before puberty when congenital hypogonadism can be diagnosed by the simple analysis of basal gonadotropin and gonadal hormone levels.

## History

The transient activity of the hypothalamus-pituitary-gonadal (HPG) axis occurring in healthy infants of both sexes has been defined as minipuberty (1). The first reports on minipuberty date back to the 1970s, but its meaning is still, in some respects, not fully understood. At birth, testosterone levels in males are in the low adult male range, while testosterone levels in females are not different from those of adult women (1, 2). In both sexes, plasma testosterone at birth is significantly higher in peripheral than in cord blood and rapidly drops within the first days of life. A second surge of testosterone occurs in male infants from the 2nd week to the 3rd month of life. After the 7th month, testosterone values are again very low, and similar to those of prepubertal boys (1). In females, testosterone levels decline soon after birth and reach the low values observed in prepubertal girls during the second week of life (2). Maternal estrogens exert an inhibitory feedback on fetal gonadotropin secretion, their removal at birth probably initiates the release of gonadotropins by the pituitary gland of the newborn, which in turn stimulates testosterone production. Early reports did not investigate estrogen secretion in female babies and most of the following studies focused on male minipuberty. Thus, the process of minipuberty in girls is not fully understood and its role in adult reproductive function is still controversial.

## Prenatal Events

### Hypothalamic-Pituitary-Gonadal (HPG) Axis in Prenatal Life: Lights and Shadows

The ontogeny of HPG axis shows peculiar characteristics including incomplete functional maturation during intrauterine and early postnatal life, functional quiescence during childhood, and a final maturation during puberty. Gonadotropin-releasing hormone (GnRH)-secreting neurons develop from the epithelium of the medial olfactory placode and migrate along nerve fibers up to the fetal hypothalamus at about 40 gestational days (3). The pituitary gland evolves and starts gonadotrophin production (follicle-stimulating hormone, FSH and luteinizing Hormone, LH) at 9 weeks of gestation (4). LH and FSH become detectable in fetal blood between 12 and 14 weeks (5). In postnatal life Kisspeptin and its receptor (KISS1R) modulate GnRH neuron activity, but early LH and FSH secretion is independent of GnRH and kisspeptin and becomes GnRH-induced only after the 30th week of gestation (6). Gonadotropin levels peak at mid-gestation, then gradually decline and appear suppressed in full-term newborns (7, 8). This pattern is probably related to the progressive increase of placental estrogens in late gestation, suppressing the activity of the fetal HPG axis (9). Overall fetal gonadotropin levels are more elevated in females than in males (5, 10), but a sexual dimorphism of gonadotropin secretion is evident (11), since LH is higher than FSH in males (12), whereas FSH predominates in females. The mid-gestational gonadotropin peak coincides with high testosterone levels and the first ovarian follicle or seminiferous tubule maturation. The relatively lower gonadotropin levels in male fetuses are probably due to the high testosterone levels, determining a partial negative feedback on their secretion (11, 12). The presence of high levels of maternally derived estrogens prevents the analysis and quantitative assessment of estrogen secretion in female fetuses.

### Lessons From Anencephaly

Early studies analyzed anencephalic human fetuses as an *in vivo* model to assess the role of HPG axis in gonadal maturation (13, 14). Anencephaly is a major congenital abnormality due to the failure of embryonic neural tube closure and cranial vault development. As a result, the brain is severely damaged by erosion. Fetuses with anencephaly are usually aborted, or die in the neonatal period. They show absent neurocranium, nearly complete destruction of cerebral hemispheres and cerebellum, protruding eyes and tongue. Different skeletal abnormalities may be associated. Hypothalamus and pituitary posterior lobe are usually absent. Anterior pituitary is generally reduced in size. Electron microscopy has shown that anterior pituitary cells contain secretory granules (14), but the release of pituitary hormones is impaired due to the absence of hypothalamic releasing factors. Baker (13) studied gonadal development in anencephalic fetuses, comparing them with control fetuses of similar gestational age. Testicular tubules of anencephalic fetuses were apparently similar to those of controls, but gonocyte number was significantly reduced. Intertubular tissue showed areas of connective tissue similar to the areas where Leydig cells were seen in control samples, but Leydig cells were markedly reduced or absent. The degree of testicular disruption was variable in different fetuses, but the absence of Leydig cells was always associated with a severe reduction or absence of the gonocytes. On the contrary, ovarian development was apparently normal in anencephalic fetuses up to about 34 weeks. Oogonia entered mitosis and increased in number, oocytes entered meiosis reaching the diplotene stage of the meiotic prophase, and normal primordial follicles appeared. Nevertheless, small antral follicles were not observed in anencephalic specimens, while they represented a typical characteristic of control ovaries after the 34th week (13). These early data suggested that hypothalamic stimulation is essential in both sexes, but in particular in male fetuses, to complete physiological gonadal cell differentiation during the last part of gestation.

### Embryogenesis and Fetal Differentiation of Male Genitalia

In 46,XY embryos, testicular differentiation starts in the gonadal ridge during the 7th gestational week, independently of gonadotrophin secretion. Somatic cells of the primordial gonad differentiate into Sertoli, peritubular, Leydig, and mesenchymal cells. Primordial germ cells stem from the endodermal layer of the yolk sac and migrate through the dorsal mesentery up to the developing gonads (15). Sertoli and germ cells aggregate and are surrounded by peritubular cells to form the primordial seminiferous cords. Mesenchymal and Leydig cells settle in the interstitial tissue. Sertoli cells produce anti-Müllerian hormone (AMH), which induces the regression of the Müllerian ducts (namely the anlagen of uterus, salpinges and upper part of the vagina). Germ cells replicate by mitosis but do not progress to meiosis during fetal life. Wolffian duct differentiate into epididymis, vas deferens, and seminal vesicle under the control of testosterone produced by Leydig cells. On the contrary, the male differentiation of the primordial urogenital sinus, and the development of prostate gland, penis and scrotum are due to diihydrotestosterone (DHT), which is the active metabolite of testosterone. Recently, an alternative pathway leading to DHT formation and not requiring testosterone has been recognized as involved in the masculinization of the human fetus. Androsterone is supposed to be the major androgen involved in this “backdoor” pathway (16). Analysis of steroid levels in different tissues has showed that androsterone is relatively high in the circulation of male fetuses during the second trimester, while it is very low in female fetuses. It does not originate from the fetal testes but is produced in the placenta and in different fetal organs, as liver and adrenal glands. The main substrate is probably the placental progesterone (16). AMH expression during embryogenesis is independent of gonadotrophin stimulation. From mid-gestation onwards, FSH stimulates Sertoli cell to replicate and produce AMH and inhibin B (15, 17). Leydig cell function during embryogenesis is modulated by human Chorionic Gonadotropin (hCG), which is later progressively replaced by LH (15). Testosterone levels begin to rise between 8 and 11 weeks of gestation, reach peak levels similar to the adult male values between 11 and 14 weeks, and progressively decline after the 20th week (9). During embryogenesis, Leydig cells produce also the protein hormone insulin-like peptide 3 (INSL3) that is involved in human testicular descent (15). INSL3 mRNA expression in fetal testis starts between the 14th and the 16th gestational week, peaks between 17 and 18 weeks, and drops around the 21st week (18). Circulating INSL3 levels between 15 and 20 weeks are 2–4 fold the prepubertal male levels (18). INSL3 binds to relaxin family peptide receptor 2 (RXFP2) on gubernacular cells, causing retraction of the gubernaculum through cell proliferation and extracellular matrix changes. This process leads to the mobilization of the testis from the initial retroperitoneal/abdominal position to the scrotum. In healthy boys, INSL3 values at 3 months of age are elevated and correlate with LH levels (19). Boys with undescended testes show significantly reduced INSL3 levels in cord blood and at 3 months of age (19). Postnatally, INSL3 is regulated by LH and implicated in Leydig cell differentiation and protection from apoptosis (18).

### Embryogenesis and Fetal Differentiation of Female Genitalia

In 46,XX embryos, AMH absence allows Müllerian ducts differentiation into salpinges, uterus, and upper portion of the vagina during the 1st trimester of gestation. On the contrary, the lower vagina, vulva, and urethra develop from the urogenital sinus (15). The development of the primordial follicles in the fetal ovaries begins just before the 13rd of gestation. The pool of primordial follicles is around 100,000 at 15 weeks, then grows speedily and reaches the number of 680,000–1,000,000 at 34 weeks. Subsequently, this pool remains stable (20) and forms the basis of female fertility. The follicular reserve develops when estrogen levels in the fetal circulation are elevated, mostly due to placental production, while the relevance of estrogen secretion by fetal ovaries is still unknown (15). Gonadotropin role in ovarian development during pregnancy is still unclear (15).

## Early Postnatal Events: Timing Of Minipuberty In Relation To Gestational Age

1) Minipuberty in healthy full-term babies

**Figure 1:**
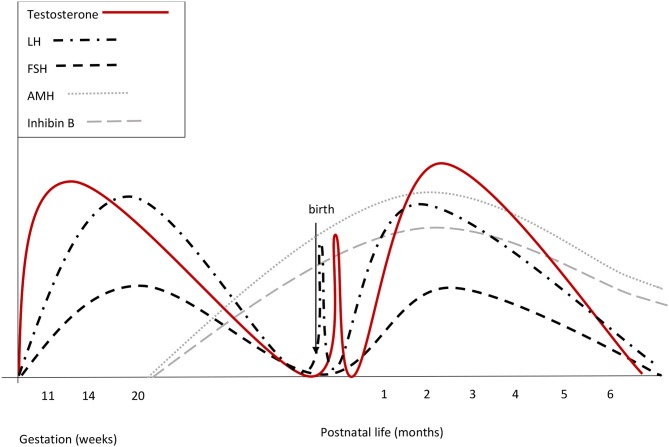
Summary of reproductive hormone changes during early life in healthy boys. Fetal gonadotrophins surge at mid-gestation, then decline and are low or undetectable in cord blood, owing to the inhibitory effect of placental estrogens. Immediately after birth, LH transiently increases by around 10-fold, followed by a testosterone peak, which lasts 12–24 h. A few days after birth, gonadotropins surge again. LH peaks between the 2nd and the 10th week of life and then gradually decreases, reaching the low prepubertal values by 6 months of age. FSH drops to the prepubertal range within the 4th month of life. Both at mid-gestation and during minipuberty, LH predominates over FSH.

**Figure 2:**
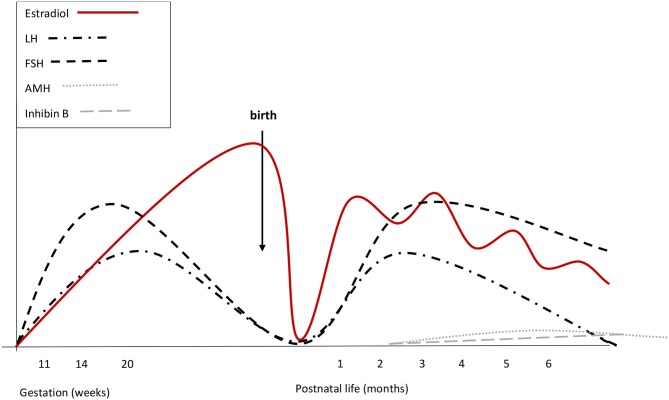
Summary of reproductive hormone changes during early life in healthy girls. Fetal gonadotrophins peak at mid-gestation. Overall gonadotropin levels are higher than in males. During fetal life placental and fetal estrogens overlap. Cord blood LH and FSH are low or undetectable, due to the inhibitory feed-back induced by placental estrogens. There are no peaks of LH or estrogens immediately after birth. Starting from the 2nd week, gonadotropins increase and stimulate estradiol secretion that remains high (although fluctuating) until at least the 6th month. LH declines at the same time as in boys, while FSH remains stably high up to 3 or 4 years of age. Both at mid-gestation and during minipuberty, FSH predominates over LH.

Cord blood LH and FSH are low or undetectable in both sexes, owing to the inhibitory feed-back induced by placental estrogens (21). A few minutes after birth, LH levels increase by around 10-fold in boys, showing a high-frequency, pulsatile secretory pattern. Gonadotropin increase is followed by a testosterone peak, which lasts 12–24 h (22). In girls, there is no peak of LH and testosterone levels are about 10-fold lower than in males (22). No peak of ovarian estrogens has been described in girls at birth. Figures 1, 2 summarize prenatal and early postnatal changes of reproductive hormones.

A few days after birth, the rapid clearance of placental estrogens from circulation causes the vanishing of the negative feedback on neonatal HPG axis and gonadotropins start to rise (23). In male infants, serum LH reaches peak values between the 2nd and the 10th week of life. Afterwards, LH gradually decreases and reaches the low prepubertal range at about 6 months of age (23, 24). The pattern of gonadotropin secretion in early infancy replicates the sexual dimorphism observed at mid-gestation, although values may show some overlap. FSH predominates in girls with a peak between 1 week and 3 months, while LH prevails in boys. LH declines in girls at the same time as in boys (23, 24). In boys, FSH decreases to the prepubertal range within the 4th month of life, while FSH remains stably high up to 3 or 4 years in girls (24).

In boys, Leydig cell number increases significantly until the 3rd month and then falls as a result of the progressive apoptosis of fetal Leydig cells (25). Testosterone secretion gradually increases with a peak between 1 and 3 months and then drops, reaching the low prepubertal levels around 6 months of age (23). Sertoli cells replicate under FSH stimulation, but they do not express androgen receptors, so they do not complete their maturation (characterized by stop of cell replication, reduction of AMH production, and blood-testicular barrier formation) and germ cells are not able to enter spermatogenesis (26). Sertoli cells secrete AMH and inhibin B. AMH reaches the highest levels at 3 months and subsequently decreases to the relatively lower and stable levels observed during childhood. In males, AMH further decreases during puberty and the average adult level is about 3% of the infant level (27). The absence of androgen receptor expression on Sertoli cells during early infancy accounts for the elevated AMH values concurrently with high testosterone levels (26). Inhibin B is already detectable in cord blood, rises soon after birth and shows peak values, higher than those observed in adults, between 3 and 6 months (23). Subsequently, inhibin B gradually decreases to the permanently low values observed throughout childhood and increases again at the onset of puberty.

From a clinical point of view, a mild increase of testicular volume is observed in the first 5 or 6 months of life, as a result of Sertoli cell proliferation, on average from 0.27 to 0.44 ml, subsequently decreasing to 0.31 ml by 9 months of age (28). This increase can be detected at ultrasound (29), although usually not evident on palpation. The high testosterone levels have a trophic effect on the external genitalia (29), penile length increases from birth to 3 months (around 1 mm/month) and pubic hair and scrotal hyperpigmentation may become evident (30).

Studies in patients with cryptorchidism associated with congenital hypogonadotropic hypogonadism have suggested that male fertility potential is defined during minipuberty (31, 32). As later discussed in this review, gonadotropins and testosterone during minipuberty induce gonocyte differentiation into type A spermatogonia, the stem cells for spermatogenesis, with the male-specific definition of cell memory and DNA methylation patterns (31, 32).

It is still unclear whether minipuberty is essential for a normal female reproductive function. Cord blood estradiol levels are elevated in both sexes, because they are almost completely of placental and maternal origin. Cord blood estrogens correlate with gestational age, twinning, mode of childbirth and pregnancy-related complications, but not with the sex of the newborn (33). During the first days after delivery, estradiol levels rapidly drop in both sexes. Starting from the 2nd week, they increase again in girls and remain high until at least the 6th month of life (34), even if wide fluctuations can be observed, probably reflecting the cycle of follicular maturation and atrophy (34). At 3 months of age, the high FSH levels stimulate granulosa cells to proliferate and produce AMH (35), and induce follicular development (35, 36). However, circulating AMH levels in females during childhood are permanently low, between 1 and 10% of those observed in males (35). Small amounts of inhibin B may be also secreted by the granulosa cells, but circulating levels are low or undetectable (34).

Uterus and mammary glands represent the main targets of estrogen action. At birth, term babies of both sexes may show palpable breast tissue, probably due to the prenatal stimulation induced by placental estrogens (37). In boys, the mammary gland gradually decreases during the first months of life, while in full-term girls palpable breast tissue commonly persists or grows, as a result of endogenous estrogen activity (38). The length of fetal uterus increases during late gestation due to placental hormones. The maximum uterine length has been reported at around 7 days of life in full-term babies, it decreases progressively during the first 3 months and then persists substantially unchanged up to the 2nd year (38).

2) Minipuberty in preterm and small for gestational age (SGA) babies

Postnatal activity of HPG axis has been reported as more pronounced and more protracted over time in preterm infants than in full-term infants. Kuiri-Hanninen et al. (29) evaluated gonadotropin and testosterone levels in serial urine samples from full-term and preterm males and compared them with testicular and penile growth. LH and testosterone levels showed similar trends in both groups, even if values detected at 7 and 30 days of life were significantly higher in premature babies, and the most premature boys showed the highest hormone levels. LH and testosterone levels fell over time in both groups, but their mean levels were still significantly different at 6 months. Higher LH and testosterone levels correlated with a more evident testicular and penile growth in preterm boys. The potential programming effects of high androgen levels in preterm infants are still unclear. Hyperandrogenism may have a programming meaning with detrimental effects on linear growth, body composition, fat distribution, glucose and lipid metabolism and blood pressure, ultimately contributing to long-term cardiovascular risk (39). During early postnatal life, brain development is still ongoing. Most axon and synapse formation occurs during the first year of life. Abnormally high testosterone levels may influence neural development, exerting “imprinting” effects on the brain of preterm boys (40).

Mean gonadotrophin levels during minipuberty in preterm girls are higher than in full-term girls and show a more protracted peak (36). The higher FSH levels have been related to ovarian immaturity, with delayed follicular development at ultrasonography and delayed onset of the negative feedback of ovarian hormones on gonadotropins. A recent study (41) showed that the high gonadotrophin levels of preterm girls decrease between 38 and 42 weeks of post-conception age, concurrently with a steep increase of estradiol, even if LH and estradiol are still positively correlated between 4 and 6 months of life. Estradiol in preterm girls increase later than testosterone in preterm boys and its peak is strongly correlated with uterus and breast growth (42). Estrogen receptor alpha is expressed in the fetal breast gland after the 30th week of gestation (43), for this reason breast development in usually absent in severely premature infants.

The ovarian hyperstimulation syndrome has been described as a clinical consequence of the intensive HPG axis activity in preterm girls (42). Sedin et al. (44) first reported this syndrome in 4 severely preterm girls at a post-conceptional age slightly preceding the expected time of delivery, as edema of the vulva, hypogastric area and thighs, associated with high estradiol levels and multiple ovarian cysts. Starzyk et al. (45) reported variably elevated estradiol concentrations (from 80 to 5,300 pmol/l) in 9 affected babies between the 35th and 39th weeks post conception. The GnRH test confirmed HPG axis activation. Breast growth (42, 46) and solitary or monthly vaginal bleeding (47, 48) have been described. The ovarian hyperstimulation syndrome has been diagnosed even before birth (49) and its pathogenesis is still not fully understood. HPG axis immaturity with incomplete development of negative feedbacks after the removal of placental steroids could be involved (45, 50).

Data on minipuberty in SGA infants are heterogeneous and conflicting. In a recent longitudinal study (51), the average urinary levels of FSH and testosterone in male infants born SGA have been described as lower than those of appropriate for gestational age (AGA) infants; in contrast, LH levels were found relatively higher. Previous studies had showed increased serum levels of FSH in SGA infants of both sexes (52) and higher plasma testosterone values in SGA boys (53). It is difficult to draw definite conclusions from these studies, as all of them included a limited number of SGA infants and used dissimilar and not comparable methods of analysis. Further controlled studies are needed to clarify whether SGA infants show distinctive hormonal patterns during minipuberty and the potential clinical implications for later life.

### Lessons From Genetic Disorders/Differences of Sex Development (DSD)

Disorders/differences of sex development (DSD) represent a heterogeneous group of congenital conditions affecting the determination and/or differentiation of chromosomal, gonadal, or anatomical sex (54). Virilized 46,XX babies, undervirilized 46,XY babies and babies with various defects of sex chromosomes may show a “different” genital appearance at birth. The sexual dimorphism of gonadotropins and gonadal hormones during minipuberty can represent a “window of opportunity” in the evaluation of a child with DSD. A recent study (55) based on a large group of healthy infants and infants with DSD aimed at the analysis of sex differences in serum levels of reproductive hormones from healthy infants, in order to define sex-specific cutoff values and to apply these cutoffs in the diagnostic workup of infants with DSD. Overall, it was confirmed that LH predominates in boys and FSH predominates in girls, even if gonadotropin levels showed a slight overlap between sexes. The LH/FSH ratio clearly differentiated males from females and the overlap was irrelevant at a cutoff level of 0.32. Inhibin B and AMH levels were remarkably higher in boys than in girls, with minimal or no overlap. AMH, LH/FSH ratio, and testosterone were the best indicators to discriminate infant sex during minipuberty. Infants with Turner syndrome, Klinefelter syndrome and 45,X/46,XY mosaicism with male phenotype, showed a LH/FSH ratio consistent with the gender of rearing. LH/FSH ratio was within the male range in infants with androgen insensitivity syndrome.

Newborns with complete androgen insensitivity syndrome (CAIS) show a female phenotype of the external genitalia, absence of Mullerian structures, blind vagina, and testes located in the inguinal canals or labia maiora. Partial androgen insensitivity (PAIS) syndrome involves a wide range of virilization defects, ranging from minimal virilization in a 46,XY individual resembling a clitoromegaly to isolated hypospadias. Mutations of the androgen receptor (AR) gene are identified in most cases, although the phenotypic expression of a given mutation may be variable (56). Low testosterone and LH values have been reported from the 1st week to the 90th day of life in patients with CAIS, while both LH and testosterone levels have been reported as “normally elevated” in infants with PAIS (57). Testosterone levels increase similarly in CAIS and PAIS after hCG administration, while peak responses of plasma LH, after stimulation with GnRH, have been described as significantly higher in infants with PAIS (57). These clear differences between CAIS and PAIS suggest that androgen action on the HPG axis is essential to stimulate LH secretion in male infants, and the fall of placental estrogen at birth is not the only factor driving the neonatal LH surge (24).

Turner syndrome has been traditionally associated with the 45,X karyotype, but it can also be related to mosaicism or structural defects of sex chromosomes, with the presence of a normal X chromosome associated with abnormalities of the other sex chromosome, X or Y (58). Incidence has been described as 1:2,130 female newborns. The phenotype is variable and may involve short stature, dysmorphic facial and body features, cardiovascular and urinary malformations. Short stature associated with gonadal dysgenesis and primary hypogonadism represents the most common clinical picture (58). Hypogonadism is the result of a massive apoptosis of the oocyte pool occurring from fetal life to late childhood (59). A retrospective longitudinal study (60) analyzed FSH and LH levels at different ages in 15 girls with Turner syndrome. Four out of 5 patients whose gonadotropins were measured during minipuberty had abnormally high FSH levels, none of them entered spontaneous puberty. The pattern of FSH secretion in these girls was biphasic: FSH was elevated during minipuberty and early childhood, dropped in mid-childhood, although rarely reached the normal prepubertal range, and began to rise again in late childhood. LH secretion was also biphasic, but only 1 girl showed remarkably high LH concentration in the first 6 months of life, while LH levels increased from late childhood to adolescence. It has been suggested that FSH measurement could be helpful for the early detection of Turner syndrome in infants with suggestive signs, especially in settings with limited resources for karyotype analysis (60).

Klinefelter syndrome is caused by the numerical chromosome aberration 47,XXY in around 80% of cases, while 20% show more severe aneuploidies (e.g., 48,XXXY) or mosaicisms (61). This condition occurs in ~1:600 newborn males and represents the most common sex chromosome disorder (62), and the most common genetic cause of hypergonadotropic hypogonadism and infertility (63). The clinical phenotype is widely variable, ranging from early testicular failure with small testes and micropenis already evident in infancy, to mild androgen deficiency and azoospermia diagnosed in adulthood (61). A few studies evaluated reproductive hormones in infants with Klinefelter syndrome and the results are conflicting. In particular, testosterone levels have been described as high, normal or decreased in different studies (64–67). Aksglaede et al. (64) analyzed serum levels of gonadotropins, inhibin B, total and free testosterone and sex hormone binding globulin (SHBG) in 10 infants with Klinefelter syndrome at 3 months of age and compared them with the values observed in a group of age-matched healthy boys. Infants with Klinefelter syndrome showed significantly higher levels of total testosterone, free testosterone, LH and FSH. Inhibin B and SHBG values did not differ from controls. FSH/inhibin B ratio was elevated when compared to controls, while LH/testosterone and LH/free testosterone ratios were similar. The high FSH/inhibin B ratio was supposed to be an early sign of Sertoli cell dysfunction, while the concurrently high testosterone and LH levels were interpreted as the result of an altered set-point of the negative feed-back induced by testosterone on gonadotropin secretion. Lahlou et al. (65) compared gonadotropin and gonadal hormone levels of 18 infants with non-mosaic Klinefelter syndrome diagnosed by antenatal testing with hormone levels of 215 healthy infants. Testosterone increased during minipuberty in infants with Klinefelter syndrome, but mean levels were lower than those of healthy infants from birth to 8 months, suggesting that an impaired Leydig cell function is already present during infancy. Conversely, LH, FSH, inhibin B and AMH levels were not different, suggesting a normal Sertoli cell function. After minipuberty, hormone levels declined to prepubertal levels in children with Klinefelter syndrome as well as in controls. An initial increase of gonadotropins, testosterone and inhibin B occurred in boys with Klinefelter syndrome at puberty, but testosterone rise stopped early and its levels ended at a low-normal adult value. Similarly, inhibin B showed a rapid decline, concurrent with the FSH rise, probably reflecting the early degeneration of seminiferous tubules (68). These conflicting findings probably reflect the remarkable heterogeneity of the clinical phenotype and the wide variability in the age of onset and progression of testicular failure in patients with Klinefelter syndrome.

### Minipuberty and Early Assessment of Hypogonadism

Cryptorchidism is evident in about 25% of infants with congenital GnRH or gonadotropin deficiency (either as isolated central hypogonadism, or in the context of combined pituitary hormone deficiencies) as compared to 1–3% of full-term infants in the general population (32). Bilateral cryptorchidism is more common than unilateral cryptorchidism in these patients and micropenis is often associated (69). These differences underscore the crucial role of minipuberty during the final stages of testicular descent and in anchoring testes securely in the scrotum (31, 32). The development of male genitalia occurs during the 1st trimester of gestation under the control of hCG. For this reason, while micropenis can be associated with cryptorchidism in congenital central hypogonadism, hypospadias is not seen (70). As already said for DSD, minipuberty is considered a key “window-of-opportunity” to identify congenital central hypogonadism (69, 71, 72).

It is noteworthy that <60% of men with congenital central hypogonadism (lacking minipuberty) achieve adult testicular volume and semen quality with gonadotropin replacement therapy (71). A plausible approach to optimize the fertility potential is to restore the hormonal milieu of minipuberty, in order to promote Sertoli and germ cell proliferation and initial differentiation (71, 72). This maturation could potentially improve the response to combined gonadotropin therapy during adolescence and adult life. Although definitive evidence is still lacking, short-term combined gonadotropin therapy (FSH+hCG/LH) in infants with absent minipuberty can stimulate normal levels of testosterone, inhibin B and AMH (72) and represent an effective alternative to testosterone for correcting micropenis (72). Gonadotrophin treatment may play an additional role facilitating (or even obviating) the surgical approach to undescended testes and may contribute to reduce surgical complications by the increase of testicular volume. Early studies investigated the role of hCG or GnRH therapy in unselected cryptorchid males (73, 74). Despite apparent success in individual patients, the possibility of unrelated spontaneous descent of retractile testes cannot be excluded in the absence of randomization and the effect does not seem sufficiently powerful to justify routine clinical use. However, targeted neonatal treatment for cryptorchid boys with absent minipuberty might reveal significantly higher success rates (75, 76). Table 1 summarizes available studies on gonadotropin treatment in order to replace minipuberty in infants with congenital hypogonadotrophic hypogonadism (77–83). In patient with congenital central hypogonadism, the impairment of HPG axis ontogeny results in a profound perturbation of germ cell-specific gene expression during late gestation with cryptorchidism at birth, and abnormal germ cell development and differentiation during minipuberty (31). Cryptorchidism-related azoospermia could be due to defects in the epigenetic pathways involved in genome stability and spermatogenesis, leading to early germ cell death. This could be the reason why early and successful surgical correction of cryptorchidism reduces, but does not eliminate the risk of infertility (32, 84).

**Table 1:** Gonadotropin treatment to replace minipuberty in infants with congenital hypogonadotrophic hypogonadism.

**References**	**Patients**	**Gonadotropin treatment**	**Basal inhibin B (ng/L)**	**Basal testicular volume (ml)**	**Inhibin B after treatment (ng/L)**	**Testicular volume after treatment (ml)**
Main et al. (77)	1	Twice weekly SC injections of rhLH 20 IU and rhFSH 21.3 IU, for 5.8 months	121	0.3	268	0.8
Bougnères et al. (78)	1	CSI of rhLH 56 IU/day and rhFSH 67 IU/day, for 4 months	167	0.6	701	2.1
	2	CSI of rhLH 50 IU/day and rhFSH 125 IU/day, for 7 months	48	0.5	426	2–1
Sarfati et al. (79)	1	CSI of rhLH 75 IU/day and rhFSH 75 IU/day, for 6 months	24	0.3	NA	2–3
Lambert and Bougneres (80)	8 patients	CSI of rhLH 50 IU/day and rhFSH 75–150 IU/day, for 5 to 6.5 months	63.3^∧^	0.43°	368^∧^	1.64°
Stoupa et al. (81)	5 patients	CSI of rhLH 75-150 IU/day and rhFSH 75 IU/day, for 3 to 6 months	95^∧^	0.7	469^∧^	2.3
Kohva et al. (82)	4 patients	rhFSH 3.4-7.5 IU/kg/week in 2-3 SC injections, for 3 to 4.5 months	76^∧^	0.12-0.15^*^	176^∧^	0.1–0.4^*^
Papadimitriou et al. (83)	10 patients	daily SC injections of rhLH 75 IU and rhFSH 150 IU, for 3 months	27.8^∧∧^	NA	365^∧∧^	1.5

*rhFSH and rhLH, recombinant human FSH and LH; SC, subcutaneous; CSI, continuous subcutaneous infusion; °measured in the 3 children whose testes were palpable in high scrotal position*;

* *volumes measured in only 2 patients*;

∧ *mean levels*;

∧∧ *median levels*.

Cryptorchidism is more common in preterm infants and spontaneous testicular descent may occur within the first 6 months of life in ~75% of cryptorchid premature infants (32). For this reason, pediatric surgeons traditionally defer formal assessment of cryptorchid neonates with the aim of avoiding unnecessary orchidopexy. This implies that the diagnostic and therapeutic window represented by minipuberty is often missed. An early systematic approach by a multidisciplinary team involving neonatologists, endocrinologists and surgeons would be desirable to assess both term and preterm infants with undescended testis (especially if bilateral and/or associated with micropenis). In these infants, a basal blood sampling between 4 and 8 weeks of life can confirm congenital hypogonadism more rapidly and with a greater accuracy than complex stimulation tests performed later in childhood (71, 72). In central hypogonadism basal LH, FSH and testosterone are very low or undetectable; while the finding of elevated gonadotropins concurrently with undetectable testosterone levels suggests congenital anorchism (vanishing testis or testicular regression syndrome). Early diagnosis of congenital hypogonadotropic hypogonadism and timely replacement therapy may contribute not only to improve the outcome of orchidopexy, but also to reduce long-term consequences of absent minipuberty.

### Minipuberty and True Puberty: Lessons From Different Forms of Central Hypogonadism

A few data concerning patients with different forms of congenital hypogonadotropic hypogonadism have suggested that minipuberty and true puberty are modulated by different mechanisms, even if they share clear similarities.

Prader–Willi syndrome (PWS) is a complex disorder related to absent expression of the paternally active genes of the chromosome region 15q11-q13 (85). Hypogonadism is one of the most relevant features and has been related both to a hypothalamic dysfunction and to a primary gonadal defect (86). Hormone levels were analyzed in a group of infants with PWS aged 1 to 3 months and compared with reference values of normal infants and prepubertal children with PWS (87). Hormone levels in infant boys with PWS were increased compared to prepubertal children and consistent with a normal minipuberty. Hormone values in infant girls with PWS did not differ significantly from the low values observed in prepubertal girls. Estradiol levels were undetectable in all but one infant girl. These data support the hypothesis that the complex hypothalamic damage of PWS may not be evident during infancy, as it progressively develops during childhood and adolescence. The reasons why HPG axis in infant girls with PWS seems to be quiescent is still unknown.

Hypogonadotropic hypogonadism due to impaired GnRH release has been related to loss-of-function mutations of KISS1R (already known as GPR54) (88). A missense homozygous KISS1R variant in (R297L) was identified in a child with micropenis, bilateral cryptorchidism and hypoplastic scrotum (89). The R297L variant reduces post-receptor signaling *in vitro*, without extinguishing it. Hormonal evaluation during minipuberty revealed very low testosterone levels (13 ng/dl), and relatively low but detectable LH levels (2.9 mIU/mL). At 11 years and 8 months, the boy entered spontaneous puberty with increasing testicular volume. By about 17 years of age, his puberty was complete and adult levels of circulating gonadotropins and testosterone were detected. This discordance suggests that GnRH neurons activity during minipuberty may depend upon the integrity of kisspeptin signaling, more than GnRH neuron functioning during true puberty.

A discordance between minipuberty and true puberty has been also documented in patients with X-linked adrenal hypoplasia congenita (AHC). X-linked AHC is due to mutations of the NR0B1 gene (nuclear receptor subfamily 0, group B, member 1) mapped on Xp21.4,5. The gene NR0B1 encodes for the nuclear receptor protein DAX1 (dosage-sensitive sex reversal, adrenal hypoplasia critical region, on chromosome X, gene 1) (90). Patients with AHC present primary adrenal failure and hypogonadotropic hypogonadism. Two independent studies (91, 92) reported an infant and his maternal uncle, both of them affected by AHC due the same NR0B1 mutation. In both studies, the uncle was affected by hypogonadotropic hypogonadism, while the infant had a normal minipuberty with appropriately elevated gonadotropin and testosterone levels.

NROB1 and KISS1R are biologically different and unrelated genes, but both of them point to the distinct mechanisms regulating minipuberty and true puberty.

### Minipuberty, Linear Growth and Body Composition

A clear sexual dimorphism of body composition is evident in human beings throughout life. Females have a significantly lower proportion of lean mass (muscles) and a higher proportion of fat mass than males. Gonadal steroids are supposed to be responsible for this dimorphism. Data about neonatal differences are conflicting, some data suggest that sex-related differences are already evident at birth (93, 94), others deny the existence of these differences (95, 96). A recent study (97) has analyzed linear growth and body composition changes using air displacement plethysmography in a large group of healthy term babies of both sexes and different ethnicities. Length, total mass, fat free mass (FFM), and percent fat mass (%FM) were evaluated between the 1st and the 3rd day of life and at 5 months. Differences were evident at both time points, with males showing greater total mass and FFM, but lower %FM. During the first months, FFM increased significantly more in males, leading to a mean difference of 410 g at 5 months. On the contrary, males showed a relatively lower increase of %FM. Length increase during the first 5 months was higher in males than in females, with a mean difference of 2.6 cm/year. A previous study (98) analyzed linear growth of 18,570 healthy infants from birth to 12 months of age, in order to characterize sex-related differences in growth velocity (GV). GV was monitored in 84 healthy infants during the first 6 months of life and compared with repeated measurements of urinary testosterone and estradiol and serum insulin-like growth factor 1 (IGF-I). IGF-1 levels did not differ significantly between sexes, while testosterone was higher in boys between 7 days and 6 months of age. GV was significantly faster from birth to 6 months of age in boys and showed a significant positive association with testosterone levels in both sexes. In particular, boys showed a significantly higher GV during the first 3 months and the most remarkable GV difference (4.1 cm/year) was observed at 1 month of age, concurrently with the postnatal testosterone surge. The differences in body composition observed ad birth are probably the result of the sex-dimorphic HPG activation during mid-gestation. Testosterone exposure during minipuberty further contributes to the definition of the sex-related physical differences that may affect health and disease later in life.

## Conclusions and Food for Thoughts

A considerable amount of information is now available about the activity of the HPG axis in the early stages of life. However, some key issues are still open and deserve further investigation:

- Hormone levels, both at mid-gestation and during minipuberty, show sex-dimorphic patterns. FSH predominates in females and LH prevails in males. Immediately after birth boys show a transient LH surge, followed by a testosterone peak lasting 12–24 h, while no LH or estradiol peaks are observed in girls. LH declines by around 6 months of age in both sexes. FSH declines nearly at the same time in males, but remains stably high up to 3 or 4 years in girls. Do these dimorphic patterns have a deterministic meaning?
- In boys, long-term testicular function and spermatogenesis seem to be “programmed” during minipuberty. Can minipuberty have a similar meaning in girls?
- Maternally-derived estrogens prevent the analysis of fetal estrogen secretion. Do estrogens from fetal ovaries affect female development?
- Postnatal activity of HPG axis has been reported as stronger and more protracted in preterm infants of both sexes. Is there a deterministic meaning in this difference? Can these distinctive features affect later life and disease risk?
- Data on minipuberty in SGA infants are scarce and conflicting. Can minipuberty in SGA babies affect later development?
- Minipuberty is defined as a diagnostic window for early detection and treatment of congenital hypogonadotropic hypogonadism, but a few data point to the distinct mechanisms regulating minipuberty and true puberty. If these data were further confirmed, would this definition be questioned?
- At birth, brain development is still ongoing and most of the axon and synapse formation is completed during the first year of life. What is the real impact of minipuberty on brain sexual imprinting? Are gonadal hormones during infancy able to affect future gender identity?
- Lastly, we now have enough data on how minipuberty begins and evolves, but what factors modulate its end and restore HPG axis quiescence until the onset of true puberty?

## Author Contributions

CB analyzed the existing data and wrote the draft. MC critically reviewed the manuscript. Both authors approved the final version of the manuscript.

### Conflict of Interest

The authors declare that the research was conducted in the absence of any commercial or financial relationships that could be construed as a potential conflict of interest. The reviewer AC declared a past co-authorship with one of the authors MC to the handling editor.
